# Proposal of a Decision-Making Model for the Provisional Restoration Alternatives in Single-Tooth Implant Treatment

**DOI:** 10.7759/cureus.45589

**Published:** 2023-09-20

**Authors:** Nurcan Deniz, Ekim Onur Orhan

**Affiliations:** 1 Department of Business Administration, Faculty of Economics and Administrative Sciences, Eskişehir Osmangazi University, Eskişehir, TUR; 2 Department of Endodontics, Faculty of Dentistry, Eskişehir Osmangazi University, Eskişehir, TUR

**Keywords:** single-tooth implant, multi-criteria decision-making, ranking, entropy, dental implant, additive ratio assessment

## Abstract

Background

The decision-making of the most appropriate provisional restoration option in single-tooth implant practice is complex under multi-criteria conditions. The aim of our study is to conduct a case study on the determination of the appropriate provisional treatment option to be used in a single-tooth dental implant interim period after placement with the help of an entropy-based additive ratio assessment.

Methodology

Eight important criteria for fulfilling this purpose have been extracted from the literature search: "esthetic potential," "patient comfort," "treatment time," "laboratory cost," "occlusal clearance," "ease of removal," "durability," and "ease of modification." Provisional treatment alternatives are "removable partial denture," "vacuum-formed appliances," "bonded extracted tooth or denture," "metal or fiber-reinforced resin-bonded fixed partial denture," "wire-retained resin-bonded fixed partial denture," "acrylic resin provisional fixed partial denture," and "implant-supported fixed provisional restoration." It has been examined which of these alternatives is most appropriate in terms of both reported specifications and artificially generated dominance scenarios. The scenarios employed are S0 (criteria are equal-weighted), S1 (the criterion is tri-fold dominant), and S2 (the criterion is two-fold dominant).

Results

"Patient comfort" was the most important criterion (wj = 0.19). The remaining criteria were ranked as "modifications," "treatment time," "durability," "esthetic potential," "laboratory cost," "occlusal clearance," and "ease of removal." The "implant-supported fixed provisional restoration" treatment option had the maximum degree of utility in the S0 (Ki = 0.782) and S2 (Ki = 0.80) categories. If "treatment time" or "occlusal clearance" is the dominant variable, "vacuum-formed appliances" had the highest degree of utility (Ki = 0.69) in S1.

Conclusions

According to the rankings and scenarios created utilizing entropy-based additive ratio assessment methods, the "implant-supported fixed provisional restoration" is the appropriate provisional option for a single-tooth implant treatment. If "treatment time" or "occlusal clearance" is an absolute criterion, the "vacuum-formed provisional appliance" will replace the appropriate option.

## Introduction

In prosthetic dentistry, temporary restorations can be used in single-tooth implant treatment, in the fabrication of fixed partial dentures, and in full-mouth implant rehabilitation. Provisional restorations are frequently used for immediate or early occlusal loading of single-tooth implants during the fabrication period of the definitive prostheses [1]. Various provisional restoration alternatives are available for optimal performance. Among these multiple alternatives, decision-making is complicated for practitioners. In addition, this problem tends to be more complex when unpredictable patient priorities such as time, esthetic potential, comfort, or durability are involved [1]. The number of treatment alternatives and the priority conditions make it hard to make a sound decision about the best option for practitioners [2]. The literature search may not always be efficient when seeking a clinical tip or a piece of brief information about the focused subject to make the best decision [3].

In the past decade, the majority of research has focused on utilizing the multi-criteria decision-making (MCDM) framework to make "sustainable" decisions in medicine and dentistry [3-12]. In complex case studies, the MCDM approaches produce objective, sustainable, analytic, and impartial decisions [2,3]. Various framework methods can be adapted to healthcare problems to evaluate ranking the alternatives or providing the best alternatives [9,10]. Specifically, case studies on material selection for dental and medical implant materials [11,12] have been conducted. Recent research developed a decision-making model and an evaluation instrument for engine-driven endodontic shaping instruments.

Even though the essential criteria for the provisional restoration options of the single-tooth implant treatment have been established [1], it remains unclear how the options are selected when priority or dominance conditions have already been established. This is also the focus of the investigation. In a recent study, the authors reported, "there is real hope for further decreases in patient harm by improving clinical decisions in the age of evidence-based dentistry and big data" [2].

The purpose of our study is to conduct a case study on the determination of the appropriate provisional treatment option to be used in a single-tooth dental implant interim period after placement with the help of entropy-based additive ratio assessment (ARAS) methods, which are in MCDM framework.

## Materials and methods

**Figure 1:**
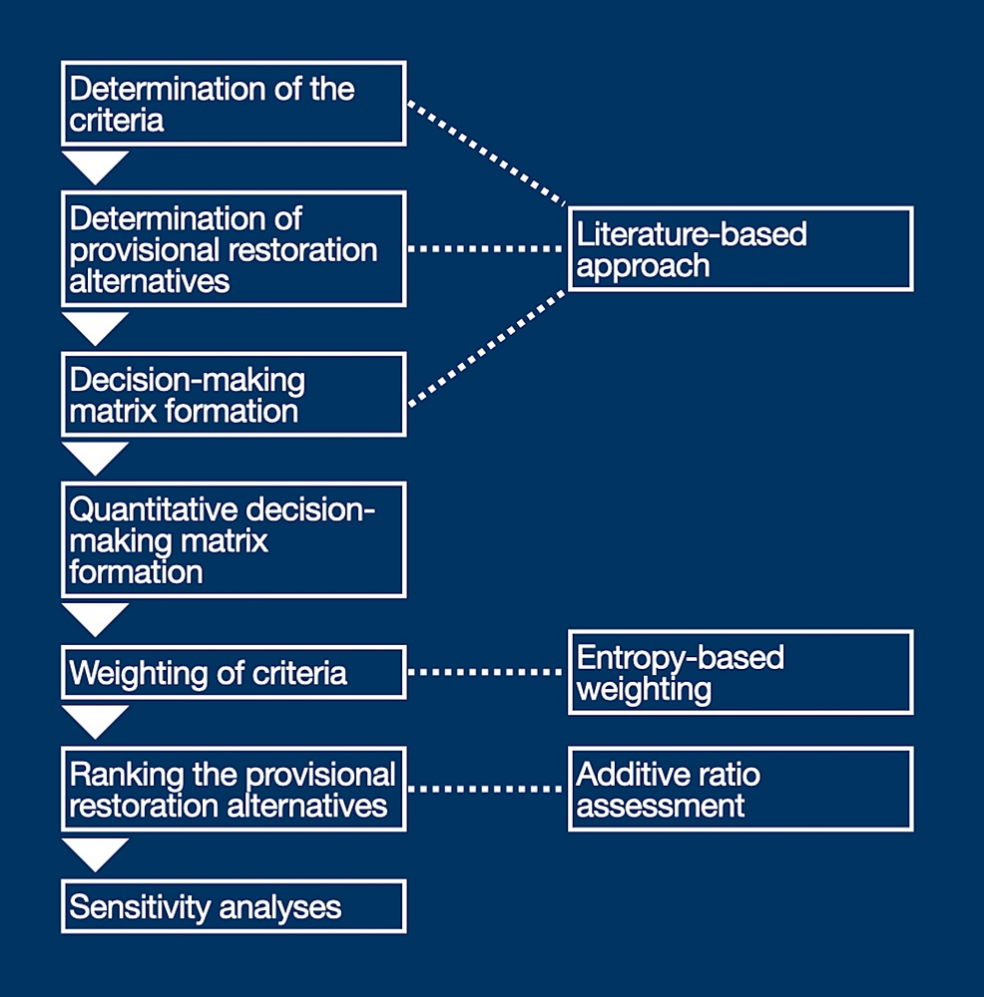
Flowchart of the methodology

Healthcare consumers are probably unfamiliar with MCDM-based frameworks [13]. Therefore, the sequential methodological details employed in this study are presented for their concession of the readers. Figure 1 displays the study design's schematic.

Data collection

In this first stage of the study's design, the provisional restoration options for single-implant treatment and their qualitative criteria were gathered using a literature-based approach. In June 2023, a literature search was performed using the search keywords "dental implant"[All Fields] AND "interim prosthesis"[All Fields] AND "provisional restoration"[All Fields] OR "provisionalization"[All Fields] in the database PubMed. Specific qualitative criteria were extracted from a previously published study [14].

Data analysis

The alternatives to provisional restorations and evaluation criteria were derived from a search of the literature in Table 1. Then, the conversion of the decision-making matrix was performed based on the criteria. Table 2 presents the conversion of the criteria to a decision-making matrix. For this purpose, the qualitative expressions were converted to numerical scales in accordance with the following rules 1-5: Rule 1: (excellent, very good, good, fair, poor) to (5, 4, 3, 2, 1); Rule 2: (lengthy, moderate, minimal) to (3, 2, 1); Rule 3: (high, medium, low, none) to (4, 3, 2, 1); Rule 4: (substantial, moderate, minimal, none) to (4, 3, 2, 1); Rule 5: (easiest, easy, moderate, difficult) to (4, 3, 2, 1).

**Table 1:** The qualitative criteria were extracted from the literature search Criterion 1: esthetic potential; Criterion 2: patient comfort; Criterion 3: treatment time; Criterion 4: laboratory cost; Criterion 5: occlusal clearance; Criterion 6: ease of removal; Criterion 7: durability; Criterion 8: modifications.

Provisional restoration options [14]	Criteria							
	Criterion 1	Criterion 2	Criterion 3	Criterion 4	Criterion 5	Criterion 6	Criterion 7	Criterion 8
Removable partial denture	Good	Poor	Minimal	Medium	Substantial	Easy	Fair	Easy
Vacuum-formed appliances	Fair	Poor	Minimal	Low	None	Easy	Fair	Moderate
Bonded extracted tooth or denture	Poor	Good	Moderate	None	Minimal	Moderate	Poor	Difficult
Metal or fiber-reinforced resin-bonded fixed partial denture	Good	Good	Lengthy	High	Moderate	Difficult	Good	Difficult
Wire-retained resin-bonded fixed partial denture	Good	Good	Moderate	Low	Moderate	Easy	Good	Moderate
Acrylic resin provisional fixed partial denture	Very good	Excellent	Lengthy	Low	Minimal	Easy	Fair	Easy
Implant-supported fixed provisional restoration	Excellent	Excellent	Lengthy	Medium	Minimal	Easy	Excellent	Easiest

**Table 2:** Decision-making matrix The underlined numbers represent the best option when the decision is made based on a single criterion in each column. Criterion 1: esthetic potential; Criterion 2: patient comfort; Criterion 3: treatment time; Criterion 4: laboratory cost; Criterion 5: occlusal clearance; Criterion 6: ease of removal; Criterion 7: durability; Criterion 8: modifications.

Options	Criteria							
	Criterion 1	Criterion 2	Criterion 3	Criterion 4	Criterion 5	Criterion 6	Criterion 7	Criterion 8
	Max	Max	Min	Min	Min	Max	Max	Max
Option 1	3	1	1	3	4	3	2	3
Option 2	2	1	1	2	1	3	2	2
Option 3	1	3	2	1	2	2	1	1
Option 4	3	3	3	4	3	1	3	1
Option 5	3	3	2	2	3	3	3	2
Option 6	4	5	3	2	2	3	2	3
Option 7	5	5	3	3	2	3	5	4

The weighting of literature-derived criteria as "esthetic potential," "patient comfort," "treatment time," "laboratory cost," "occlusal clearance," "ease of removal," "durability," and "ease of modification" was made using a validated mathematical theory of communication called Shannon’s entropy [15]. In addition to using entropy outputs, the sensitivity analysis of the literature-derived data was also conducted using outputs from the entropy algorithm. The initial sensitivity analysis of this study weighed each criterion equally (S0). Each criterion was deemed to have a 0.125 weighting factor. In addition, two distinct scenarios were developed to simulate the importance or dominance of each criterion. For this determination, each criterion was theoretically assigned a specific importance level. In scenarios I and II, the dominance levels were set at 3 and 2, respectively. In scenario 1 (S1), it was supposed that the dominant criterion was three times more dominant than the rest. Accordingly, a weight of 0.30 was assigned to the predominate criterion and 0.10 to the remainder. In scenario 2 (S2), it was assumed that the importance or level of dominance of the dominant criterion was two-fold greater than the remainder. Accordingly, the dominant criterion of S2 and S1 was allocated a 0.22 and 0.11 weighting, respectively. To clarify the entropy method, the stepwise theoretical equations are summarized below.

The entropy theory was employed to assess the weights of each criterion. According to the theory, the following equations 1 to 4 were performed using inputs. The initial step is normalization with Equation 1. The purpose of this step is to eliminate anomalies associated with various units and scales. *pij* indicates the normalized value of the *ith* option for the *jth* criterion. *m* indicates the number of options.

$p_{ij} = \frac{xij}{\sum_{i=1}^{m}xij}$ (1)

The second step involves evaluating the entropy of each criterion (ej) using Equation 2.

$e_{j}=-\frac{1}{lnm}\left ( \sum_{i=1}^{m} pijlnpij\right)$ (2)

The third step involves calculating the dispersion (dj) for each criterion Equation 3.

$d_{j} = \left | 1 - e_{j} \right |$ (3)

The fourth step is determining the weight for each criterion (wj) using Equation 4.

$w{_{j}} = \frac{d}{\sum_{i=1}^{m}dj}$ (4)

Using entropy, a preliminary ranking of provisional restoration options was generated. In addition, sensitivity analyses were conducted to investigate the impact of varying criterion weights on the ranking of provisional restoration options. After determining the weight for each criterion, ARAS was used to rank the provisional restoration options in descending order. To clarify the ARAS method, the stepwise theoretical equations are summarized below.

The optimal value of each criterion was characterized by ARAS. To define the optimum value of each criterion, the following equations were employed. The initial step was matrix formation. Each *Xij* value within the matrix represents the performance value of the *ith* alternative according to the *jth* criterion Equation 5.

$X_{0j} = i_{min}x_{ij}$, if preferable (5)

The second step involved normalization. If the direction of the criterion was oriented as maxima, the $\overline{X}ij$ normalized decision matrix consisting of $\overline{X}ij$ values was computed by employing Equation 6.

$\overline{Xij} = \frac{Xij}{\sum_{i=0}^{m}Xij}$ (6)

If the direction of the criterion goes minima, the decision matrix consists of two sub-steps as follows: Equations 7.1 and 7.2.

$X^{*}ij = \frac{1}{Xij}$ (7.1)

$\overline{Xij} = \frac{X^{*}ij}{\sum_{i=0}^{m}X^{*}ij}$ (7.2)

The third step involved the addition of weighting for criteria (wj). Each matrix value in the normalized decision-making matrix was multiplied by the weights to create a normalized-weighted matrix. Each weight was calculated as 0 wj 1, and the sum of all weights is 1. The $\widehat{X}ij$ values in this matrix were computed using Equation 8.

$\widehat{X}ij = \overline{X}ij\cdot Wj$ (8)

The fourth step involved determining the values of the optimality function. *Si* represented the value of the optimality function for the *ith* option, as computed by Equation 9.

$S_{i} = \sum_{j=1}^{n} \cdot \widehat{X}ij \cdot i =0,1,...m$ (9)

Equation 10 was used to determine the utility degree (Ki) for each opinion, and S0 represented the optimal function value.

$K_{i} = \frac{S_{i}}{S_{0}}i = 1,2,...m$ (10)

## Results

The entropy of each criterion is listed in Table 3. Accordingly, "patient comfort" was weighted as the most important criterion (Wj = 0.19). The remaining criteria were ranked as "modifications," "treatment time," "durability," "esthetic potential," "laboratory cost," "occlusal clearance," and "ease of removal."

**Table 3:** Results of entropy-based weighting per each criterion Criterion 1: esthetic potential; Criterion 2: patient comfort; Criterion 3: treatment time; Criterion 4: laboratory cost; Criterion 5: occlusal clearance; Criterion 6: ease of removal; Criterion 7: durability; Criterion 8: modifications. e_j_: entropy of each criterion; d_j_: dispersion of each criterion; w_j_: weight of each criterion. Parameters are detailed under the method heading.

Parameter	Criterion 1	Criterion 2	Criterion 3	Criterion 4	Criterion 5	Criterion 6	Criterion 7	Criterion 8
e_j_	0.96	0.93	0.95	0.97	0.97	0.98	0.95	0.95
d_j_	0.04	0.07	0.05	0.03	0.03	0.02	0.05	0.05
w_j_	0.12	0.19	0.14	0.09	0.09	0.07	0.14	0.15

Rankings of the options per weighting methods are listed in Table 4. There was no difference in rankings of the options between entropy and equal weighting. In the entropy-based ARAS, "implant-supported fixed provisional restoration" had the maximum degree of utility in the S0 (Ki = 0.782). The remaining utility degrees were ranked as follows: "acrylic resin fixed partial denture" (Ki = 0.649), "vacuum-formed appliances" (Ki = 0.565), "wire-retained resin-bonded fixed partial denture" (Ki = 0.552), "removable partial denture" (Ki = 0.528), "bonded extracted tooth or denture" (Ki = 0.463), and "metal or fiber-reinforced resin-bonded fixed partial denture" (Ki = 0.433).

**Table 4:** Rankings of the options per weighting methods Si shows the optimality function value for the corresponding option. Ki shows the "utility degree" of an option, which is an interval value ranging from 0 to 1, and shows the relative efficiency. *Ki is the ordered form of the corresponding Ki values (descending order).

Entropy-based simple additive weighting					Equal weighting				
Options	Si	Ki	Ranking		Options	Si	Ki	Ranking	
	0.201		Options	Ki*		0.200		Options	Ki*
Option 1	0.106	0.528	Option 7	0.782	Option 1	0.108	0.542	Option 7	0.751
Option 2	0.114	0.565	Option 6	0.649	Option 2	0.123	0.616	Option 6	0.638
Option 3	0.093	0.463	Option 2	0.565	Option 3	0.098	0.490	Option 2	0.616
Option 4	0.087	0.433	Option 5	0.552	Option 4	0.082	0.412	Option 5	0.559
Option 5	0.111	0.552	Option 1	0.528	Option 5	0.112	0.559	Option 1	0.542
Option 6	0.131	0.649	Option 3	0.463	Option 6	0.127	0.638	Option 3	0.490
Option 7	0.157	0.782	Option 4	0.433	Option 7	0.150	0.751	Option 4	0.412

Rankings of the dominance scenarios per criteria are listed in Table 5. The S0 rankings overlapped with the S2, having differences in utility degree values. In S1, options were ranked as different from S0 and S2. When "treatment time" or "occlusal clearance" was three-fold more dominant than the remaining criteria, the highest degree of utility was assigned "vacuum-formed appliances" (Ki = 0.69) in S1.

**Table 5:** Results of additive ratio assessments of dominant scenarios "A" shows each restoration option. A1: Provisional removable partial denture; A2: Vacuum-formed appliances; A3: Bonded extracted tooth or denture; A4: Metal or fiber-reinforced resin-bonded fixed partial denture; A5: Wire-retained resin-bonded fixed partial denture; A6: Acrylic resin provisional fixed partial denture; A7: Implant-supported fixed provisional restoration. Criterion 1: esthetic potential; Criterion 2: patient comfort; Criterion 3: treatment time; Criterion 4: laboratory cost; Criterion 5: occlusal clearance; Criterion 6: ease of removal; Criterion 7: durability; Criterion 8: modifications. "S" shows the scenario. Ki shows the "utility degree" of an option, which is an interval value ranging from 0 to 1, and shows the relative efficiency.

Criterion 1		Criterion 2		Criterion 3		Criterion 4		Criterion 5		Criterion 6		Criterion 7		Criterion 8	
S1 (Ki)	S2 (Ki)	S1 (Ki)	S2 (Ki)	S1 (Ki)	S2 (Ki)	S1 (Ki)	S2 (Ki)	S1 (Ki)	S2 (Ki)	S1 (Ki)	S2 (Ki)	S1 (Ki)	S2 (Ki)	S1 (Ki)	S2 (Ki)
A7 (0.80)	A7 (0.78)	A7 (0.80)	A7 (0.78)	A2 (0.69)	A7 (0.71)	A7 (0.66)	A7 (0.70)	A2 (0.70)	A7 (0.72)	A7 (0.79)	A7 (0.77)	A7 (0.80)	A7 (0.78)	A7 (0.80)	A7 (0.78)
A6 (0.67)	A6 (0.66)	A6 (0.71)	A6 (0.68)	A7 (0.69)	A2 (0.66)	A6 (0.61)	A6 (0.62)	A7 (0.69)	A2 (0.66)	A6 (0.69)	A6 (0.67)	A6 (0.59)	A6 (0.61)	A6 (0.66)	A6 (0.65)
A2 (0.57)	A2 (0.59)	A5 (0.57)	A2 (0.57)	A1 (0.63)	A6 (0.60)	A3 (0.60)	A2 (0.60)	A6 (0.61)	A6 (0.62)	A2 (0.68)	A2 (0.65)	A2 (0.57)	A2 (0.59)	A2 (0.59)	A2 (0.60)
A5 (0.56)	A5 (0.56)	A2 (0.54)	A5 (0.56)	A6 (0.58)	A1 (0.59)	A2 (0.59)	A3 (0.55)	A5 (0.51)	A5 (0.53)	A5 (0.63)	A5 (0.59)	A5 (0.57)	A5 (0.56)	A1 (0.59)	A1 (0.57)
A1 (0.55)	A1 (0.55)	A3 (0.51)	A1 (0.51)	A5 (0.55)	A5 (0.55)	A5 (0.55)	A5 (0.55)	A3 (0.49)	A1 (0.51)	A1 (0.61)	A1 (0.58)	A1 (0.51)	A1 (0.53)	A5 (0.55)	A5 (0.55)
A4 (0.44)	A3 (0.45)	A1 (0.47)	A3 (0.50)	A3 (0.49)	A3 (0.49)	A1 (0.49)	A1 (0.51)	A1 (0.47)	A3 (0.49)	A3 (0.51)	A3 (0.50)	A4 (0.45)	A3 (0.45)	A3 (0.44)	A3 (0.46)
A3 (0.43)	A4 (0.43)	A4 (0.44)	A4 (0.43)	A4 (0.39)	A4 (0.40)	A4 (0.37)	A4 (0.39)	A4 (0.39)	A4 (0.40)	A4 (0.40)	A4 (0.40)	A3 (0.42)	A4 (0.43)	A4 (0.37)	A4 (0.39)

**Figure 2:**
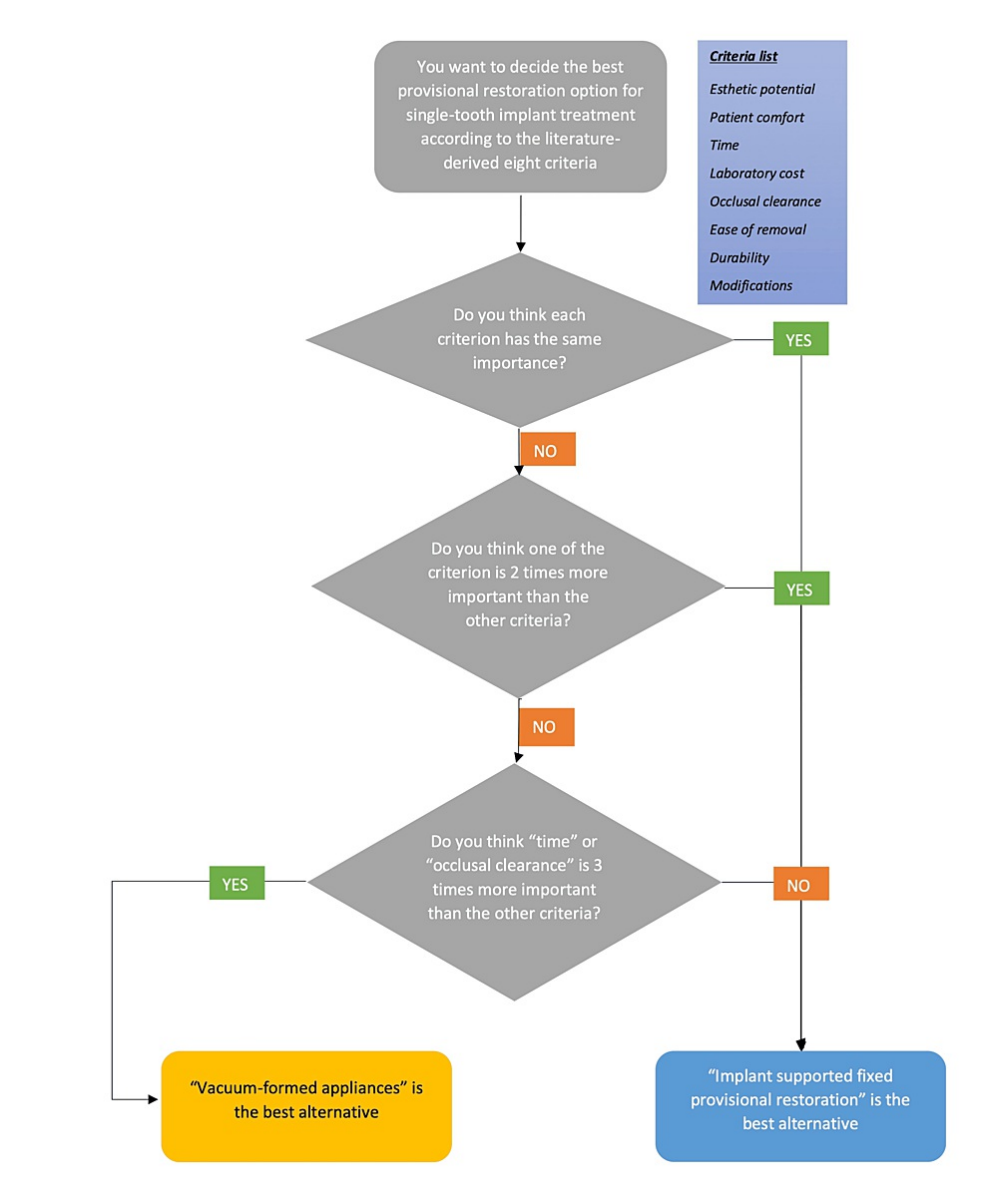
A flowchart illustration was added to summarize the study outcomes for clinicians

A flowchart illustration was added to summarize the study outcomes for clinicians in Figure 2 and was produced from the results of sensitivity analysis to increase the understandability of data presentation in Tables 1-5. It needs to be taken into consideration that there are numerous scenarios based on the number of criteria, number of treatment options, and importance degree of decision makers for them. Figure 2 only shows the results of the tested weights of literature-based criteria and the best options from the literature-based treatment list.

## Discussion

The decision-making of the most appropriate provisional restoration option in single-tooth implant practice is complex under multi-criteria conditions. To assist the dental professionals regarding the case problem, this study generated literature-based rankings per different clinical scenarios.

The "treatment selection problem" was elaborated with a conceptual framework developed that was based on MCDM in this methodology. It is possible that readers who work in the healthcare industry are unfamiliar with the MCDM-based frameworks [13]. As a result, the proposed methodology for this study is also discussed in the discussion section for better understanding. The hypothesized criteria that were derived throughout the literature search in this study were collected and converted into a matrix that was used in the decision-making process. In this investigation, the entropy method was preferred in weighting calculations due to its high objectivity for each criterion [15,16].

The rankings were then constructed based on the various dominance configurations. As a decision-making aid in clinical practice, the findings provide objective, systematically generated rankings of the available treatment options. If a general practitioner or specialist seeks to adapt the associated literature-based suggestions to a clinical case, the generated rankings can promptly assist as a decision-making tool. In agreement with the relevant literature, the principal advantage of systematic decision-making is to permit decision-making among numerous identical cases or clinical scenarios with uniformity, accuracy, and consistency [8].

According to our findings, "patient comfort" (wj = 0.19 in the entropy) was the most prominent criterion. On the other hand, "implant-supported fixed provisional restoration" was rated as the first option regardless of the criteria in S0. Likely, fixed provisional restorations for single-implant treatment have also been suggested due to presenting better soft tissue integration, patient comfort, and esthetics [14,17-21]. Our findings are in agreement with the previous studies [22-24]. Furthermore, the theoretically generated dominance hypotheses (S1 and S2) were also consistent with previous studies [14,17-24].

One unique circumstance appeared only in S1. Consequently, if "treatment time" or "occlusal clearance" is three times more influential than the remaining criteria, "vacuum-formed appliances" had the highest degree of utility (Ki = 0.69). This finding suggests that if treatment time is dominant in a clinical case, vacuum-formed provisional appliances may be the best option for provisional restorations due to their rapid formation and low cost of production [14,22,23]. These devices can be manufactured quickly in a laboratory or dental facility [22]. In addition, vacuum-formed appliances stabilize adjacent teeth against rotational forces and preserve the surgical site postoperatively [14,23,24]. The esthetic consequence of these devices has been compared to that of a removable provisional partial denture [14]. However, vacuum-formed appliances are less comfortable than alternative fixed provisional restorations [14]. This study's determination that "patient comfort" is the most essential criterion may be a significant factor in the low degree of utility of vacuum-formed provisional appliances.

It did not take into account the replacement of a primary temporary restoration with the new one in the scenarios. Thus, if the antecedent provisional restoration must be replaced with the secondary provisional restoration, the weighting of the criteria must be modified [15,16]. The weighting of the criterion "laboratory cost" could shift from medium to "high" in this instance. However, no published information is available for comparing the costs of repeated provisional restoration applications. This cannot be transcribed to a sensitivity scenario. Although simple entropy-based additive weighting and additive ratio evaluation methods can be helpful in the selection of the most suitable temporary restorations, one of the most important reasons for making temporary restorations in the peri-implant mucosa, particularly in the anterior region, is to optimize the proximal contact location relative to the adjacent natural tooth or prosthetic crown and establish peri-implant mucosal margins in harmony with the gingival contours of the adjacent teeth. Consequently, one of the most significant limitations of our study is that the selection of appropriate provisional restoration in clinical practice may vary by patient. The authors also noted that the study did not consist of "the replacement of primary temporary restorations with new ones" subjects due to a gap in the literature, which could impact the weighting of specific criteria.

The MCDM-based approach presents objective decision-making for solving complex or multivariable problems, selecting materials, and generating rankings or hierarchies. Many reports are available in the literature on which the MCDM framework was utilized to solve various case problems in health care [10,15,25,26] and dental care [3,27]. This treatment selection case was studied with the MCDM framework to provide objective decision-making and avoid bias. The literature-derived qualitative suggestions and criteria were converted to the data source of this study. However, the literature-derived criteria should be considered questionable. The considered criteria might have been reported from clinical experiences or observations of the experts regarding this case. Hence, further prospective studies on the case may validate our findings. The clinicians can add new criteria to the same decision-making problem. Also, the presented scenarios could be reproduced with various inputs of weightings or dominance levels of the criteria in further study designs. In addition, criteria weights could be determined using subjective methods based on expert decisions such as the analytic hierarchy process (AHP). The other MCDM techniques could be used to rank the treatment options and can be compared as well.

To solve numerous complex and multicriteria case problems in the dental care industry, future study designs should incorporate MCDM frameworks more often.

## Conclusions

Based on the constraints of the MCDM case study, it is feasible to draw the following conclusions: for the first time, a quantitative evaluation was conducted on literature-based provisional restoration possibilities, which were extracted and assigned weights. Additionally, clinical dominance situations that were relevant were generated and then associated based on clinical criteria. Based on the rankings and scenarios generated by the implementation of entropy-based additive ratio assessment techniques, it can be concluded that the most suitable provisional alternative for a single-tooth implant treatment is the "implant-supported fixed provisional restoration." If the absolute criteria for treatment duration or occlusal clearance are met, the vacuum-formed temporary appliance will be selected as the suitable choice. The findings of this research have the potential to provide dental practitioners with valuable and unbiased information to support their clinical decision-making in the relevant area.
